# The tumor microenvironment: a dynamic ecosystem and therapeutic nexus in modern oncology

**DOI:** 10.3389/fphar.2026.1836055

**Published:** 2026-06-09

**Authors:** Meiqi Zhang, Yao Lu, Xingxing Yuan

**Affiliations:** 1 Department of Graduate School, Heilongjiang Academy of Traditional Chinese Medicine, Harbin, China; 2 First Clinical Medical College, Heilongjiang University of Chinese Medicine, Harbin, China; 3 Department of Gastroenterology, Heilongjiang Academy of Traditional Chinese Medicine, Harbin, China

**Keywords:** cancer-associated fibroblasts, immunotherapy, metabolic reprogramming, nanomedicine, tumor microenvironment, tumor-associated macrophages

## Abstract

The tumor microenvironment (TME) has emerged as a central orchestrator of carcinogenesis, therapeutic resistance, and immune evasion, fundamentally reshaping the understanding of cancer as an ecosystem disease rather than a cell-autonomous genetic disorder. This review synthesizes contemporary advances in deconstructing the cellular and acellular architecture of the TME, encompassing cancer-associated fibroblasts, tumor-associated macrophages, aberrant vasculature, and a dynamically remodeled extracellular matrix. The molecular underpinnings of TME-mediated pathogenesis are critically evaluated, including metabolic reprogramming, epigenetic dysregulation, and systemic microbiome crosstalk, which collectively enforce immunosuppression and drive adaptive resistance. Building on this mechanistic framework, a new generation of therapeutic strategies designed to reprogram this malignant niche is highlighted: precision nanotechnologies for targeted and stimuli-responsive delivery; next-generation immunotherapies such as logic-gated CAR-T cells, bispecific engagers, and oncolytic viruses; metabolic and epigenetic modulators; stromal and vascular normalization approaches; and microbiome-based interventions, for instance fecal microbiota transplantation and defined bacterial consortia. Transformative tools, including patient-derived organoids, tumor-on-a-chip systems, 3D bioprinting, and artificial intelligence-powered multi-omics, are now enabling predictive modeling and personalized therapeutic forecasting. Despite persistent challenges posed by intratumoral heterogeneity, cellular plasticity, and the complexity of combination trial design, the convergence of these multidisciplinary approaches provides an unprecedented toolkit to durably reprogram the TME. Mastering this dynamic ecosystem is paramount to overcoming therapeutic roadblocks, and the strategic integration of these advances heralds a definitive paradigm shift toward TME-centric, adaptive, and personalized cancer therapy.

## 1 Introduction

The classical view of cancer as a disease driven solely by the accumulation of genetic mutations within tumor cells has been fundamentally and irrevocably redefined over the past 2 decades. It is now unequivocal that tumor progression, metastatic dissemination, and therapeutic response are dictated not in isolation, but by a complex, adaptive, and co-evolving ecosystem, the tumor microenvironment (TME) (Sun S. et al., 2025). This paradigm shift recognizes the malignancy as an organ-like structure, where the neoplastic cells are but one component within a heterogeneous assemblage of non-cancerous cellular constituents. These include a diverse array of immune cells (lymphocytes, macrophages, myeloid-derived suppressor cells (MDSCs)), cancer-associated fibroblasts (CAFs), endothelial cells, and pericytes, all embedded within a dynamically remodeled extracellular matrix (ECM) and bathed in a unique biochemical milieu characterized by hypoxia, acidosis, and nutrient scarcity (García-Caballero et al., 2022; Liu et al., 2025). This recognition has elevated the TME from a passive bystander to a central orchestrator of malignancy.

Crucially, this microenvironment is not a static scaffold but a dynamic entity engaged in continuous, reciprocal crosstalk with tumor cells. Through a complex network of cytokines, chemokines, growth factors, and metabolic byproducts, signals from the TME actively drive the acquisition of the classic hallmarks of cancer: sustaining proliferative signaling, evading growth suppressors, resisting cell death, enabling replicative immortality, inducing angiogenesis, and activating invasion and metastasis (Yuan et al., 2024). Furthermore, the TME is a master regulator of the emerging hallmarks of immune evasion and phenotypic plasticity, which directly underpin therapy resistance and disease relapse (Feng et al., 2026; Zhou et al., 2022). For instance, immunosuppressive cells like regulatory T cells (Tregs) and M2-polarized tumor-associated macrophages (TAMs) create a barrier to anti-tumor immunity, while the hypoxic, acidic niche promotes epigenetic and metabolic adaptations that enhance tumor cell survival and stemness (Meng et al., 2025; Wang L. et al., 2025).

This ecological understanding explains the frequent failure of therapies targeting only tumor cells. Conventional chemotherapy and radiation can inadvertently select for resistant clones and, through damaging stromal elements, foster a more immunosuppressive and pro-fibrotic TME, ultimately promoting recurrence (Garemilla et al., 2025; Darragh and Karam, 2026). Targeted therapies, while initially effective, frequently succumb to adaptive resistance mechanisms orchestrated by the stromal compartment, which provides alternative survival signals to tumor cells (Shaik et al., 2026). Even revolutionary immunotherapies like immune checkpoint inhibitors (ICIs) fail in a majority of patients, often due to a “cold” or immune-excluded TME landscape that lacks cytotoxic T cell infiltration or is dominated by immunosuppressive cells and signals (Ouyang et al., 2024).

Consequently, the modern oncological frontier has decisively shifted towards holistically understanding and therapeutically reprogramming the TME. This review consolidates the latest advancements across this vast and interdisciplinary landscape. We will first deconstruct the TME’s core cellular and acellular components, detailing their origins, heterogeneity, and pro-tumorigenic functions. Subsequently, we will explore cutting-edge diagnostic and *ex vivo* modeling technologies, such as patient-derived organoids and tumor-on-a-chip systems, that are essential for deciphering TME complexity. The core of the review will detail the armamentarium of therapeutic strategies; from nanomedicine and epigenetic modulators to next-generation immunotherapies and microbiome engineering, designed to target and reprogram the TME. We culminate in a critical discussion of the challenges in clinical translation, including intratumoral heterogeneity, adaptive resistance, and biomarker development. The synthesis of insights from recent works aims to provide a foundational resource for researchers and clinicians dedicated to advancing TME-centric cancer therapy, positing that mastering this malignant ecosystem is the key to overcoming therapeutic roadblocks and achieving durable cures.

## 2 Deconstructing the tumor microenvironment

### 2.1 Cancer-associated fibroblasts

CAFs are arguably the most abundant and influential stromal cell population within many solid tumors, particularly in carcinomas characterized by dense desmoplasia, such as pancreatic, breast, and colorectal cancers. They are not a uniform entity but a highly heterogeneous population originating from multiple sources. Resident tissue fibroblasts are the most common precursors, activated by tumor-derived signals like TGF-β and PDGF. Other sources include mesenchymal stem cells (MSCs) recruited from the bone marrow, stellate cells which are notably involved in cancers such as those of the liver and pancreas, and even local endothelial or epithelial cells that undergo endothelial-mesenchymal transition (EndMT) or epithelial-mesenchymal transition (EMT) in response to chronic inflammation and hypoxia. This diverse ontogeny contributes significantly to their functional heterogeneity.

Advanced single-cell transcriptomic and proteomic analyses have moved beyond the generic “CAF” label, delineating distinct subtypes with specialized and often divergent roles in tumor progression (Elyada et al., 2019; Nurmik et al., 2020; Paraskeva et al., 2026). These subtypes exist on a dynamic spectrum and can interconvert based on environmental cues.

#### 2.1.1 Myofibroblastic CAFs (myCAFs)

Typically found in close proximity to tumor cells, myCAFs are characterized by high expression of alpha-smooth muscle actin (α-SMA), fibroblast activation protein (FAP), and PDGFRβ. Their primary function is the massive deposition, contraction, and cross-linking of ECM components, primarily collagen I and III, leading to the formation of a dense, stiff, and fibrotic stromal barrier (Zhou et al., 2025). This desmoplastic reaction, while initially perhaps an attempt to wall off the tumor, ultimately promotes cancer cell invasion by providing “tracks” for migration and generating biomechanical forces that activate pro-invasion signaling. Moreover, this dense ECM acts as a significant physical and biochemical barrier, impeding the penetration of chemotherapeutic drugs and the infiltration of immune effector cells, a major contributor to therapy resistance.

#### 2.1.2 Inflammatory CAFs (iCAFs)

This subtype is often located farther from the tumor epithelium, in regions rich in inflammatory signals. iCAFs are defined by their secretory phenotype, producing a plethora of cytokines and chemokines such as interleukin-6 (IL-6), leukemia inhibitory factor (LIF), and CXCL12 (Elyada et al., 2019; Rovers et al., 2025). Their function is predominantly paracrine: IL-6/JAK/STAT3 signaling promotes cancer cell stemness and proliferation; CXCL12 mediates the recruitment of immunosuppressive cells and directly inhibits T cell activity; and LIF supports tumor cell survival. iCAFs are thus key instigators of a chronic, tumor-promoting inflammatory state.

#### 2.1.3 Antigen-presenting CAFs (apCAFs)

A more recently identified subset, apCAFs express major histocompatibility complex class II (MHC-II) molecules and the invariant chain CD74 (Zhu et al., 2026). While they can present antigen, they generally lack the necessary co-stimulatory molecules, such as CD80 and CD86, required for effective T cell activation. This suggests apCAFs may engage CD4^+^ T cells in a non-productive or even tolerogenic manner, potentially inducing anergy or driving the differentiation of regulatory T cells (Tregs), thereby contributing to immune suppression.

#### 2.1.4 Metabolic CAFs (meCAFs)

Reflecting the intense metabolic demands of the TME, this subtype undergoes profound metabolic reprogramming. MeCAFs exhibit high glycolytic flux and express monocarboxylate transporter 4 (MCT4) to export lactate, pyruvate, and ketone bodies (Mitchell et al., 2021). Through a process termed the “reverse Warburg effect,” these metabolites are then imported by adjacent cancer cells via the MCT1 to fuel their mitochondrial oxidative phosphorylation (OXPHOS) and anabolic pathways, effectively acting as metabolic feeders for the tumor (Limbu et al., 2025; Liang et al., 2026; Linderman et al., 2025).

### 2.2 Tumor-associated macrophages

Macrophages are innate immune cells with remarkable plasticity, capable of adopting a spectrum of activation states. In the TME, they are predominantly polarized towards an M2-like, alternatively activated phenotype, earning the name tumor-associated macrophages (TAMs). They are recruited in large numbers by tumor-derived chemoattractants such as CCL2, CSF-1, and VEGF (Galon and Bruni, 2019). Once embedded, TAMs become key mediators of almost every stage of tumor progression and a cornerstone of therapy resistance. Their pro-tumor functions are multifaceted: TAMs are potent promoters of neovascularization. They secrete VEGF, PDGF, and matrix metalloproteinase 9 (MMP9), which collectively stimulate the sprouting, survival, and abnormal maturation of new blood vessels, resulting in the chaotic and leaky tumor vasculature (Chen P. et al., 2025). TAMs are architects of the immunosuppressive milieu. They express immune checkpoint ligands like PD-L1, secrete anti-inflammatory cytokines such as IL-10 and TGF-β, and produce enzymes that metabolically suppress T cell function. A key mechanism is the upregulation of arginase-1 (ARG1), which depletes local L-arginine, an essential nutrient for T cell proliferation and function (Martinenaite et al., 2025). They also sequester iron, further restricting T cell activity.

Therapeutic strategies targeting TAMs are therefore a major focus. These include: depleting them using inhibitors of the CSF-1/CSF-1R axis; blocking their recruitment via CCR2/CCL2 antagonists; and, most promisingly, reprogramming them from an M2 to an M1-like, anti-tumor phenotype. Reprogramming can be achieved with CD40 agonists, Toll-like receptor (TLR) agonists, or nanoparticle-delivered agents that alter their metabolic and signaling pathways (Li H. et al., 2026; Yang et al., 2023; Xu J. et al., 2025).

### 2.3 The tumor vasculature

The tumor vasculature, formed by tumor-associated endothelial cells (TECs), is structurally and functionally abnormal. Driven by an excess of pro-angiogenic signals such as VEGF over anti-angiogenic factors, it is characterized by excessive branching, irregular diameters, incomplete pericyte coverage, and disrupted basement membranes (Leone et al., 2024). This results in a hyperpermeable, chaotic network. The consequences of this aberrant vasculature are profound. It leads to heterogeneous blood flow, causing regions of severe hypoxia and acidosis. The leakiness also increases interstitial fluid pressure, which, combined with the dense ECM, creates a formidable barrier to the delivery of systemically administered therapeutics. Furthermore, the abnormal endothelial walls and aberrant expression of adhesion molecules impede the extravasation and infiltration of immune effector cells into the tumor parenchyma. Interestingly, TECs themselves undergo metabolic reprogramming, shifting towards glycolysis and fatty acid synthesis to support their rapid, dysfunctional proliferation, which further disturbs vascular function (Zhan et al., 2026).

The therapeutic strategy has evolved from indiscriminate vessel destruction, or anti-angiogenesis, to the concept of vascular normalization. The goal is to use specific, low-dose regimens of anti-angiogenic agents, for example, bevacizumab and certain tyrosine kinase inhibitors, to transiently “prune” the excess, immature vessels and fortify the remaining ones. This normalization phase reduces hypoxia and interstitial pressure, improves perfusion, and enhances the delivery and efficacy of both chemotherapy and immunotherapy (Baruch et al., 2021; Tang et al., 2025). Timing and dosing are critical, as excessive inhibition can revert the vasculature to a dysfunctional state.

### 2.4 The immune landscape

The tumor immune microenvironment (TIME) is a dynamic battlefield between anti-tumor immunity and pro-tumor immunosuppression. The anti-tumor forces include cytotoxic CD8^+^ T cells, T helper 1 (Th1) CD4^+^ T cells, natural killer (NK) cells, and M1-like macrophages. However, in most established tumors, their function is potently suppressed by a coordinated network of immunosuppressive elements.

#### 2.4.1 Regulatory T cells

These specialized CD4^+^ T cells are crucial for maintaining self-tolerance but are co-opted by the tumor. They suppress effector T cell function through multiple contact-dependent and soluble mechanisms: expressing CTLA-4 to outcompete CD28 on effector T cells; consuming local IL-2 via their high-affinity IL-2 receptor; and producing immunosuppressive metabolites. A key pathway involves the ectoenzymes CD39 and CD73, which convert extracellular ATP to adenosine, a potent suppressor of T cell and NK cell activity (Mersich et al., 2025; Shen J. et al., 2025).

#### 2.4.2 Myeloid-derived suppressor cells

This heterogeneous population of immature myeloid cells expands dramatically in cancer. They suppress T and NK cell function via the production of ARG1 and inducible nitric oxide synthase (iNOS), which deplete arginine and produce reactive nitrogen species. They also generate reactive oxygen species (ROS) and sequester essential nutrients like cysteine, creating a metabolically hostile niche for lymphocytes (Zhan et al., 2026; Jou et al., 2024).

#### 2.4.3 Immunosuppressive metabolites

The TME is awash with metabolites that inhibit immune cell function. Lactate, a byproduct of glycolysis metabolism, suppresses the activity of cytotoxic T cells and NK cells. Adenosine, generated through the sequential activity of the ectoenzymes CD39 and CD73, engages immunosuppressive A2A receptors on the surface of immune cells. Another key metabolite, kynurenine, is produced via the catabolism by the enzyme indoleamine 2,3-dioxygenase 1, commonly known as IDO1. Kynurenine activates the aryl hydrocarbon receptor (AhR) to drive Treg differentiation and impair effector T cells function (Wang et al., 2026; Yang Q. et al., 2025; Li and Xiao, 2025). The spatial organization of these cells is diagnostically and prognostically critical. Tumors are often classified into three distinct immunophenotypes: the immune-inflamed phenotype, characterized by T cells present within the tumor parenchyma; the immune-excluded phenotype, where T cells are trapped at the stromal border; and the immune-desert phenotype, marked by a general paucity of T cells throughout the tumor (Jin et al., 2025; Chen M. et al., 2025). This classification scheme strongly predicts clinical response to immunotherapy.

### 2.5 The extracellular matrix and exosomes

**FIGURE 1:**
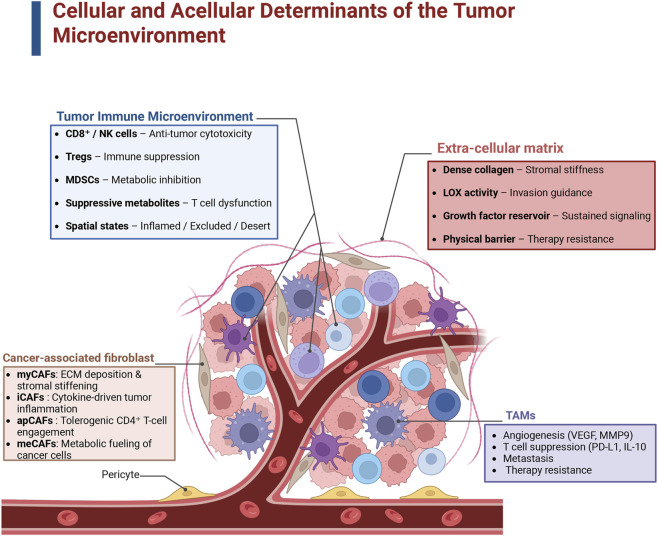
Cellular and acellular determinants of the tumor microenvironment.

The acellular components of the TME are equally active participants. The ECM undergoes dramatic remodeling, primarily driven by CAFs. It becomes stiffer, more cross-linked through the action of enzymes such as lysyl oxidase (LOX), and its composition shifts. Beyond providing mere structural support, the remodeled ECM acts as a reservoir for growth factors including VEGF and TGF-β, modulates integrin-mediated survival and migration signals, and presents a physical barrier that hinders drug penetration and immune cell motility (Shen T. et al., 2025). Exosomes and other extracellular vesicles (EVs) have emerged as critical long-range communication vehicles. These nanometer-sized lipid bilayers are shed by all cells in the TME, especially tumor cells. They carry a complex cargo of proteins, lipids, DNA, and diverse RNA species such as miRNAs, lncRNAs, and circRNAs. Tumor-derived exosomes can reprogram recipient cells: they transfer oncogenic signals to normal cells, educate bone marrow progenitors to prepare distant organs for metastasis, thereby establishing the pre-metastatic niche, suppress immune cell function by delivering PD-L1 or inhibitory miRNAs, and confer therapy resistance by exporting drugs or pro-survival signals (Hussain et al., 2025; Yang L. et al., 2025). Notably, hypoxia dramatically alters exosomal cargo, enriching for miRNAs like miR-210 that promote angiogenesis, metastasis, and treatment resistance in recipient cells (Salehi-Sangani et al., 2025). Targeting exosome biogenesis, release, or uptake is an emerging therapeutic strategy to disrupt this systemic communication network Figure 1.

Schematic representation of the tumor microenvironment (TME) highlighting key cellular and acellular components that collectively promote tumor progression and therapy resistance. Cancer-associated fibroblasts (CAFs) are depicted as a heterogeneous stromal population with distinct functional states, including extracellular matrix (ECM) remodeling, cytokine-driven inflammation, immune tolerance, and metabolic support of cancer cells. Tumor-associated macrophages (TAMs) adopt predominantly pro-tumorigenic roles, driving angiogenesis, immune suppression, metastasis, and resistance to therapy. The tumor immune microenvironment comprises both anti-tumor effector cells (CD8^+^ T cells and NK cells) and immunosuppressive elements such as regulatory T cells (Tregs), myeloid-derived suppressor cells (MDSCs), and inhibitory metabolites, with spatial immune states influencing therapeutic responsiveness. The remodeled ECM contributes to stromal stiffness, invasion guidance, sustained growth factor signaling, and physical barriers to immune cell infiltration and drug delivery. Together, these components form an integrated, co-opted ecosystem that supports malignant growth.

## 3 Molecular mechanisms of TME-mediated pathogenesis

The cellular and acellular components of the TME do not act in isolation. Their collective pro-tumorigenic function is orchestrated through a series of fundamental molecular mechanisms that reprogram cellular behavior, enforce immunosuppression, and drive adaptive resistance. Understanding these core pathways; metabolic reprogramming, epigenetic dysregulation, and systemic microbiome crosstalk, is essential to developing strategies to dismantle the tumor’s supportive ecosystem.

### 3.1 Metabolic reprogramming and nutrient competition

The TME is a metabolically restrictive and fiercely competitive ecosystem. Tumor cells, immune cells, and stromal cells vie for limited nutrients such as glucose, glutamine, and amino acids, engaging in a metabolic tug-of-war that tumor cells and immunosuppressive elements are typically rigged to win (Wang et al., 2026). This metabolic reprogramming is not a passive response to hypoxia but an active, signal-driven process that sustains tumor growth and blunts anti-tumor immunity.

#### 3.1.1 The reverse warburg effect and metabolic coupling

One of the most illustrative examples of stromal-tumor metabolic symbiosis is the Reverse Warburg Effect. In this paradigm, CAFs are induced by ROS from tumor cells to undergo aerobic glycolysis, even in the presence of oxygen. They upregulate MCT4 to export the resulting lactate and pyruvate into the extracellular space. Adjacent cancer cells, which often upregulate MCT1, avidly import these metabolites to fuel their own mitochondrial oxidative phosphorylation (OXPHOS) and anabolic pathways (Niveau et al., 2025; Xu Z. et al., 2025). This metabolic coupling allows cancer cells to perform efficient OXPHOS while outsourcing the glycolytic burden, optimizing energy production and biomass synthesis. This relationship extends to other metabolites; CAFs also undergo autophagy and glutaminolysis to secrete amino acids like alanine and glutamine, further feeding the tumor’s biosynthetic demands (Chen P. et al., 2025).

#### 3.1.2 Nutrient scavenging and immune cell starvation

Immunosuppressive cells actively deplete nutrients essential for effector T cell function. MDSCs and some tumor cells highly express ARG1, which depletes L-arginine from the microenvironment. T cells deprived of arginine exhibit reduced T-cell receptor (TCR) expression and proliferative capacity, leading to cell cycle arrest (Zhan et al., 2026; Cai et al., 2025). Similarly, the enzyme IDO1, expressed by dendritic cells and tumor cells, catabolizes the essential amino acid tryptophan into kynurenine. Tryptophan starvation activates the integrated stress response in T cells, while kynurenine itself acts as an immunomodulatory metabolite by activating the AhR, promoting Treg differentiation and driving T cell exhaustion (Yang Q. et al., 2025).

#### 3.1.3 Lipid metabolism as a functional switch

Altered lipid metabolism plays a crucial role in shaping the TME. Tregs within tumors preferentially utilize fatty acid oxidation (FAO) for their energy needs, which is intrinsically linked to their stable suppressive phenotype. Inhibiting this metabolic pathway can impair Treg function, presenting a targetable vulnerability to relieve immunosuppression (Bakir et al., 2025). Conversely, cancer cells and CAFs can engage in *de novo* lipogenesis or scavenge lipids to support membrane synthesis, signaling molecules, and energy storage. Specific CAF subtypes, such as meCAFs, exhibit a rewired lipid metabolism that supports tumor growth, and inhibiting key enzymes like CPT1A in these fibroblasts can disrupt tumor progression (Zhu et al., 2026).

#### 3.1.4 Oncometabolite signaling

Metabolic waste products are not merely waste; they are potent signaling molecules. Lactate, once considered a terminal glycolysis byproduct, is now recognized as a key oncometabolite. High extracellular lactate levels, characteristic of the TME, can suppress NK and cytotoxic T cell function. Lactate also signals through the G-protein coupled receptor GPR81 on tumor and stromal cells, promoting angiogenesis, inducing M2 macrophage polarization, and stabilizing hypoxia-inducible factor 1-alpha (HIF-1α) to further drive adaptive responses (Zhang S. et al., 2026). This creates a vicious cycle where glycolysis-driven acidosis and lactate production reinforce the immunosuppressive and pro-angiogenic features of the TME.

### 3.2 Epigenetic dysregulation and cellular plasticity

Epigenetic mechanisms; including DNA methylation, histone modifications, and chromatin remodeling, are highly dynamic in the TME and serve as a central interface between environmental cues and cellular phenotype. They are fundamental to the plasticity of tumor and stromal cells, enabling them to adapt, evade therapy, and persist.

#### 3.2.1 Driver of phenotypic plasticity

Key transitions in tumor biology, such as EMT, acquisition of stem-like properties, and transdifferentiation into other cell types, are governed by epigenetic reprogramming. Signals from the TME, such as TGF-β, hypoxia via HIFs, and inflammatory cytokines, activate or repress epigenetic modifiers like EZH2, histone methyltransferase (HDACs), and DNA methyltransferases (DNMTs) (Sherif et al., 2025). These modifiers reshape the chromatin landscape, silencing epithelial genes while activating mesenchymal or stemness programs, allowing tumor cells to become more invasive, resistant, and capable of seeding metastases.

#### 3.2.2 Architect of immune evasion

Tumors can epigenetically silence genes critical for immune recognition, creating an “immunologically cold” microenvironment. This includes hypermethylation of promoters for tumor-associated antigens (TAAs) and components of the antigen-presenting machinery (APM), such as HLA class I genes (Panda et al., 2024). By downregulating these molecules, tumor cells become invisible to cytotoxic T cells. Furthermore, epigenetic regulators can control the expression of immune checkpoint ligands like PD-L1, allowing dynamic immune evasion in response to therapy.

#### 3.2.3 Generation of drug-tolerant persister cells

A major cause of relapse is the survival of a small subpopulation of drug-tolerant persister (DTP) cells. These cells are not genetically mutant but have entered a reversible, slow-cycling state driven by profound epigenetic adaptations. These changes, often involving histone demethylation or chromatin compaction, allow them to survive the initial therapeutic insult. Upon withdrawal of therapy, these persister cells can regenerate the tumor, often with acquired genetic resistance (Wang Z. et al., 2025). Targeting these epigenetic states is a strategy to eradicate the root of relapse.

#### 3.2.4 Viral mimicry as a therapeutic opportunity

Interestingly, epigenetic drugs can turn the tumor’s defenses against itself. DNA methyltransferase inhibitors like azacitidine and decitabine can induce “viral mimicry” by demethylating and reactivating endogenous retroviral (ERV) sequences embedded in the genome (Laranjeira et al., 2023). The resulting double-stranded RNA structures are sensed as viral infection by cytosolic sensors like MDA5, triggering a robust type I interferon (IFN) response. This inflames the TME, enhances tumor immunogenicity by upregulating antigen presentation, and can re-sensitize “cold” tumors to immune checkpoint blockade, providing a powerful rationale for combining epigenetic therapy with immunotherapy.

### 3.3 The gut-tumor axis

The influence of the TME extends beyond the local tumor site to include systemic physiological systems, most notably the gut microbiome. A growing body of evidence establishes the gut microbiota as a key modulator of anti-tumor immunity and response to therapy, particularly immunotherapy (Wei et al., 2026; Yu et al., 2026).

#### 3.3.1 Mechanisms of microbial influence

Specific bacterial taxa and their metabolites can systemically shape the immune landscape of distant tumors. Beneficial metabolites include short-chain fatty acids (SCFAs) like butyrate, produced by fiber-fermenting bacteria. Butyrate acts as an HDAC inhibitor in immune cells, enhancing the anti-tumor activity of CD8^+^ T cells and promoting the development of dendritic cells with a greater capacity for antigen presentation (Lu et al., 2024). Other metabolites, such as inosine, can enhance Th1 cell differentiation and the efficacy of ICIs, while certain secondary bile acids can modulate Treg activity.

#### 3.3.2 Clinical impact and dysbiosis

The composition of the gut microbiome has been correlated with clinical outcomes. Patients with a more diverse microbiome enriched in specific “favorable” taxa (*Akkermansia muciniphila*, *Faecalibacterium prausnitzii*) demonstrate significantly better responses to ICIs in cancers like melanoma and non-small cell lung cancer. Conversely, dysbiosis (microbial imbalance) or the use of broad-spectrum antibiotics, which deplete these communities, is strongly associated with primary and secondary resistance to ICIs (Yoshinami et al., 2025).

#### 3.3.3 Therapeutic modulation via microbiota transplantation

The causal role of the microbiome has been demonstrated through fecal microbiota transplantation (FMT). In clinical studies, transferring fecal microbiota from patients who responded to ICIs into non-responding patients with refractory melanoma could re-sensitize a subset of these patients to the therapy, leading to tumor regression (Yang et al., 2026; Pamungkas et al., 2025). This proof-of-concept highlights the therapeutic potential of modulating the gut microbiome using approaches such as FMT, probiotics, or prebiotics, to overcome resistance and improve the efficacy of TME-targeting therapies.

**FIGURE 2:**
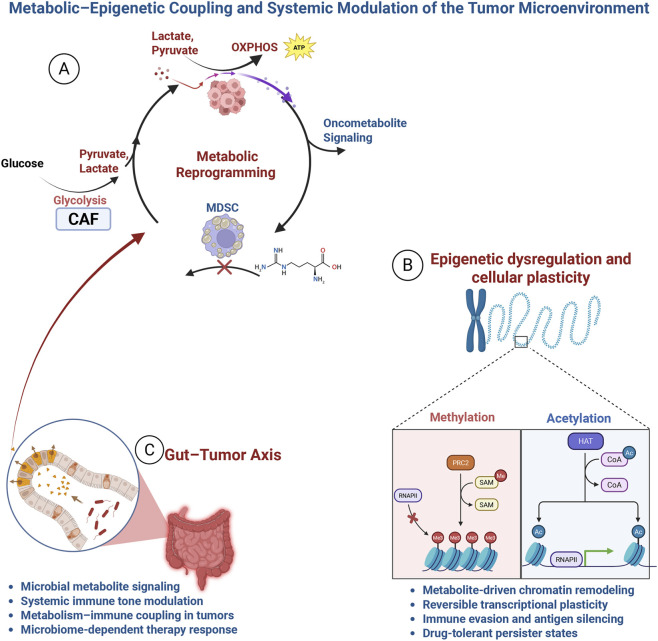
Metabolic reprogramming, epigenetic dysregulation, and systemic microbiome-derived signals cooperatively shape the tumor microenvironment. **(A)** *Metabolic reprogramming within the TME is driven by coordinated interactions between malignant, stromal, and immune cells. CAFs undergo glycolysis and export lactate and pyruvate, which are utilized by tumor cells to fuel OXPHOS. Immunosuppressive myeloid cells, including MDSCs, deplete key nutrients, contributing to immune cell dysfunction. Accumulating metabolites act as oncometabolites, reinforcing tumor-supportive signaling networks*. **(B)** *Metabolic cues directly influence epigenetic states in tumor cells. Altered metabolite availability drives chromatin remodeling through changes in DNA methylation and histone acetylation, enabling reversible transcriptional plasticity. These epigenetic adaptations promote immune evasion, antigen silencing, and the emergence of drug-tolerant persister cell states*. **(C)** *The gut--tumor axis acts as a systemic modulator of the TME. Microbiome-derived metabolites enter circulation and indirectly tune tumor metabolism and immune tone, thereby influencing metabolic--immune coupling within tumors and shaping therapeutic responsiveness. Together, these interconnected mechanisms converge to establish and maintain a pro-tumorigenic microenvironment.*

Together, these molecular mechanisms; metabolic, epigenetic, and systemic, illustrate the depth and complexity of the TME’s control over tumor fate. They reveal a network of dependencies and vulnerabilities that, when targeted in concert, offer a promising path to disrupt the tumor’s supportive niche and restore anti-tumor immunity Figure 2.

## 4 Frontiers in modeling and analyzing the TME

Traditional two-dimensional (2D) monolayer cell cultures, while useful for foundational studies, fundamentally fail to recapitulate the intricate spatial organization, cellular heterogeneity, and dynamic biochemical gradients of the TME. This disconnect has historically limited the predictive value of preclinical research. To bridge this translational gap, a suite of advanced experimental and analytical technologies has emerged, enabling researchers to deconstruct the TME’s complexity with unprecedented fidelity. These frontiers in modeling and analysis are not merely incremental improvements but revolutionary platforms that provide actionable insights for precision oncology.

### 4.1 Patient-derived organoids

Patient-derived organoids (PDOs) are three-dimensional (3D) microtissues grown from primary tumor samples or biopsies. Their principal strength lies in their ability to retain the genetic, phenotypic, and cellular heterogeneity of the patient’s original tumor, including epithelial cancer cells, and in some advanced cultures, resident stromal elements (Li R. et al., 2026). PDOs can be banked and expanded, serving as a renewable, biologically relevant avatar for the patient’s disease. Their primary application is in personalized drug screening, where a panel of therapies can be tested on a patient’s own organoids to identify the most effective treatment regimen, a step towards functional precision medicine (Ma et al., 2024). Beyond monotherapy testing, PDOs are increasingly used to model tumor-stroma interactions and the evolution of therapy resistance under selective pressure. A significant advancement is the development of immune-organoid co-cultures, where PDOs are co-cultured with autologous or allogeneic immune cells, such as tumor-infiltrating lymphocytes and CAR-T cells. This system allows for the direct study of tumor-immune cell interactions, the evaluation of immunotherapy efficacy, and the investigation of mechanisms of immune evasion in a controlled yet patient-specific context (Wu et al., 2022).

### 4.2 Organ-on-a-chip and tumor-on-a-chip

While organoids excel at capturing cellular composition, organ-on-a-chip technology adds the critical dimension of dynamic physiological flow. These are microfluidic devices fabricated from biocompatible polymers, containing miniature channels lined with living cells that mimic the structure and function of human tissues. Tumor-on-a-chip systems specifically engineer key TME features that are absent in static cultures: controlled perfusion mimicking blood flow, shear stress on endothelial cells, and stable oxygen and nutrient gradients that generate hypoxic cores (Poljak et al., 2026; Lipreri et al., 2025). This enables real-time, high-resolution investigation of processes central to cancer biology. Researchers can visually monitor angiogenesis; the sprouting of new blood vessels, in response to pro- or anti-angiogenic factors. They can track immune cell trafficking, observing how T cells or neutrophils adhere to, extravasate through, and migrate within an engineered tumor stroma. Furthermore, these platforms are ideal for studying drug penetration and distribution, providing quantifiable data on how the physical TME barrier affects therapeutic efficacy. By integrating multiple tissue types, such as tumor, endothelium, and stroma components, on interconnected chips, more complex models of metastatic seeding or organ-specific interactions are being developed.

### 4.3 3D bioprinting

3D bioprinting takes tissue engineering a step further by offering precise spatial control over the TME’s architecture. Using automated dispensing systems, bio-inks; hydrogels laden with living cells, including tumor cells, CAFs, immune cells, and endothelial cells, are deposited layer-by-layer to build complex, pre-defined 3D structures (Ruchika et al., 2024). This technology allows researchers to dictate the location of different cell types and to tailor the composition and stiffness of the bio-ink, thereby accurately mimicking the physical properties of the native ECM. One can, for instance, print a ductal structure surrounded by a defined stroma, or create a gradient of stromal density to study its impact on invasion. The primary application is in building bespoke, reproducible tumor models for high-throughput drug testing and immunotherapy screening. It holds particular promise for creating models of rare tumor subtypes or for incorporating a patient’s own cells in a geometrically controlled manner to build personalized test platforms.

### 4.4 Advanced ‘omics and artificial intelligence: decoding complexity

The sheer cellular and molecular complexity of the TME requires powerful analytical tools. Single-cell RNA sequencing (scRNA-seq) has been transformative, moving beyond bulk tissue analysis to deconvolute cellular heterogeneity at unprecedented resolution. It can identify rare cell populations, define novel cellular states such as CAF or TAM subtypes, and infer intercellular communication networks by analyzing ligand-receptor pairs (Sun Y. et al., 2025). Spatial transcriptomics adds a crucial layer by preserving the geographical context of this data. Technologies like 10x Genomics Visium or multiplexed error-robust fluorescence *in situ* hybridization (MERFISH) map gene expression directly within the tissue architecture, revealing how cellular function varies between the invasive front, the tumor core, or perivascular niches (Lin et al., 2025).

The volume and multidimensionality of data generated from these ‘omics platforms, combined with digital pathology images, are integrated and interpreted using artificial intelligence (AI) and machine learning. AI algorithms can identify complex, non-linear patterns within the TME data that are invisible to the human eye. Applications include: predicting patient prognosis and therapy response by correlating spatial immune signatures with clinical outcomes (Jin et al., 2025; Ozkerim et al., 2025); identifying novel therapeutic targets within the stromal compartment by analyzing differential gene expression networks (Khan et al., 2025); and simulating TME dynamics *in silico* to test hypotheses about intervention strategies before moving to costly and time-consuming wet-lab experiments (Ruan et al., 2025). The convergence of high-fidelity models and high-dimensional analytics is creating a virtuous cycle, where models generate rich data for AI, and AI guides the design of more sophisticated, physiologically relevant models. Together, these frontiers are providing an unparalleled toolkit to dissect the TME, moving the field from descriptive observation to predictive, mechanistic understanding.

## 5 Therapeutic strategies to reprogram the tumor microenvironment

The profound understanding of the TME as a dynamic orchestrator of malignancy has catalyzed a paradigm shift in cancer therapy. Moving beyond direct tumor cell killing, the new frontier focuses on reprogramming, altering the function of stromal and immune components to dismantle the tumor’s support system, restore anti-tumor immunity, and sensitize the ecosystem to conventional treatments. This section details the multifaceted armamentarium of strategies under development, ranging from nano-scale engineering to systemic microbiome modulation.

### 5.1 Nanotechnology for precision TME targeting

Nanocarriers, typically 1–200 nm in size, are engineered systems designed to overcome the formidable biological barriers of the TME and achieve spatiotemporally controlled delivery of therapeutic agents. Their utility lies in their multifunctional potential: they can be designed for targeting, responsive release, combination delivery, and diagnostic imaging.

#### 5.1.1 Targeting and stimuli-responsive release

Passive targeting exploits the enhanced permeation and retention (EPR) effect, a phenomenon enabled by the leaky vasculature and poor lymphatic drainage of tumors which allows nanoparticles to accumulate. Active targeting significantly enhances specificity by decorating the nanoparticle surface with ligands such as antibodies, peptides, and aptamers. These ligands bind to receptors that are overexpressed on tumor cells, for example. The EGFR and HER2 receptors on tumor cells or the fibroblast activation protein, FAP, which is expressed on cancer-associated fibroblasts. To further minimize off-target effects, researchers engineer “smart” nanocarriers to release their payload only in response to TME-specific stimuli. Key stimuli include the low pH of the tumor interstitium, overexpressed enzymes like MMPs or cathepsins, and the high redox potential, commonly exploited via disulfide bonds that are cleaved by the elevated glutathione levels found in this environment (Deng et al., 2025). This ensures drug release is concentrated within the TME.

#### 5.1.2 Long-term biocompatibility and safety considerations

While nanotechnology offers tremendous therapeutic potential, the long-term biocompatibility and potential pro-inflammatory effects of nanocarriers require careful evaluation. Accumulation of non-biodegradable nanoparticles in reticuloendothelial organs such as the liver and spleen can lead to chronic inflammation and foreign body reactions (Egbuna et al., 2021). Additionally, certain nanocarriers may trigger complement activation-related pseudoallergy (CARPA), manifesting as hypersensitivity reactions upon intravenous administration. Surface charge, size, and shape significantly influence these effects; cationic nanoparticles, for instance, tend to induce greater inflammatory responses than anionic or neutral counterparts. Strategies to mitigate these risks include the use of biodegradable polymers like PLGA and polylactic acid, PEGylation to reduce immunogenicity, incorporation of anti-inflammatory moieties, and careful optimization of dosing schedules to allow clearance between administrations (Samir et al., 2022). Regulatory guidelines for nanomedicine are evolving to require comprehensive biocompatibility and immunotoxicity assessment prior to clinical translation.

#### 5.1.3 Multifunctional and immunomodulatory platforms

Nanosystems are being designed as theranostic platforms that combine therapy and imaging. For instance, manganese dioxide (MnO_2_) nanoparticles can catalytically convert tumor-overexpressed hydrogen peroxide (H_2_O_2_) into oxygen, thereby alleviating hypoxia to improve radiotherapy and immunotherapy, while simultaneously serving as a contrast agent for magnetic resonance imaging (MRI) (Guo et al., 2025). In immunotherapy, nano-adjuvants are a breakthrough. A prominent example is a system that co-delivers an agonist of the cGAS-STING pathway, such as a cyclic dinucleotide, along with manganese ions (Mn^2+^). This combination synergistically potentiate dendritic cell activation and subsequent T cell priming, effectively converting immunologically “cold” tumors into “hot” one (Sun G. et al., 2025). Furthermore, biomimetic nanoparticles, such as those coated with hybrid membranes from cancer cells and immune cells, combine homotypic tumor-targeting capability with the ability to engage and activate the immune system (Issaka and Amu-Darko, 2025).

#### 5.1.4 Nanotechnology-enabled gene editing

The delivery of macromolecular genetic tools like CRISPR-Cas9 represents a major challenge. Lipid nanoparticles (LNPs) and polymeric vectors are being optimized to deliver CRISPR components specifically to TME cells. This opens the possibility of editing the ecosystem, such as knocking out the PD-L1 gene in tumor cells to enhance immune recognition, silencing TGF-β in CAFs to reduce immunosuppression, or disrupting pro-angiogenic genes in endothelial cells (Rauf et al., 2025).

### 5.2 Immunotherapy combinations to overcome the immunosuppressive TME

The redundancy of immunosuppressive pathways within the TME renders monotherapies, particularly single-agent immune checkpoint blockade, frequently ineffective. Therefore, rational, mechanism-based combinations are essential to dismantle this coordinated resistance network.

#### 5.2.1 Next-generation checkpoint inhibitors

To overcome resistance to PD-1/PD-L1 blockade, intense efforts are focused on developing inhibitors targeting alternative T cell co-inhibitory receptors. Key targets include LAG-3, TIGIT, TIM-3, and VISTA. These pathways are frequently upregulated in the TME following initial PD-1 pathway inhibition, leading to persistent T cell exhaustion. Consequently, dual blockade strategies, such as combining anti-PD-1 with anti-LAG-3 antibodies, aims to provide more comprehensive T cell reinvigoration (Luo et al., 2026).

#### 5.2.2 Engineered adoptive cell therapies

While revolutionary in hematological cancers, CAR-T cells face significant barriers within the solid TME. A central strategy to overcome this is engineering “armored” CAR-T cells. This involves the co-expression of various functional modules: cytokines like IL-7, IL-15 to enhance T cell persistence; dominant-negative TGF-β receptors to neutralize this potent immunosuppressant; chemokine receptors matchiong those secreted by the tumor, such as CCR2 or CXCR2, to improve infiltration; and switch receptors that convert an inhibitory signal, for instance from PD-1, into a costimulatory one (Wu et al., 2026; Zhang Z. et al., 2026).

#### 5.2.3 Bispecific T Cell engagers

Bispecific T cell engagers are antibody-based constructs designed with one binding site for a tumor-associated antigen and another for the CD3 complex on T cells. This physically redirects polyclonal T cells to kill tumor cells irrespective of their native TCR specificity. Their application in solid tumors is challenged by on-target, off-tumor toxicity and the immunosuppressive TME. Innovative solutions involve developing conditionally active bispecific T cell engagers that are activated only within the TME, as well as exploring localized delivery to minimize systemic exposure and toxicity (Xiong et al., 2026).

#### 5.2.4 Oncolytic viruses

Genetically engineered oncolytic viruses (OVs), derived from strains such as herpes simplex or vaccinia, serve as multipronged tool for TME-reprogramming. They selectively replicate in and lyse cancer cells, inducing immunogenic cell death (ICD) and releasing a broad of tumor antigens and danger-associated molecular patterns. These viruses can be further armed with transgenes encoding immune-stimulating cytokines like GM-CSF, immune checkpoint blockers, or T cell engagers. This creates a potent *in situ* vaccination effect, inflaming immunologically “cold” tumors and rendering them more susceptible to combination with systemic ICIs (Xu J. et al., 2025; Li J. et al., 2026; Tang et al., 2026).

#### 5.2.5 Rational combination logic

The selection of combination partners is strategically guided by the need to overcome specific, complementary resistance mechanisms. A prime example is the combination of PD-1 inhibitors with anti-angiogenic agents like bevacizumab, which aims to transiently normalize vasculature. This reduces hypoxia, and improve T cell infiltration of cytotoxic T cells (Rovers et al., 2025). Another rational strategy pairs ICIs with epigenetic modulators, such as DNMT or HDAC inhibitors, to enhance tumor immunogenicity via mechanisms like viral mimicry and to impair the function of immunosuppressive cells (Goyal et al., 2023; Wang H. et al., 2025; Xu Y. et al., 2025). Furthermore, the synergy between radiation and ICIs leverages radiation-induced ICD to act as an *in situ* vaccine, priming a systemic anti-tumor immune response that checkpoint blockade can then amplify (Yijun et al., 2025).

#### 5.2.6 Strategies for converting cold tumors to hot tumors

The presence of an immunosuppressive, or “cold” TME; characterized by low T cell infiltration, high levels of immunosuppressive cells such as Tregs and MDSCs, and dense physical barriers, constitutes a major cause of resistance to ICIs. Converting these immunologically quiescent tumors into inflamed, “hot,” tumors is therefore a central goal of modern immuno-oncology (Ruan et al., 2025). Multiple complementary strategies have emerged:

OVs represent a powerful immunotherapeutic approach. These genetically engineered agents selectively replicate in and lyse cancer cells, thereby inducing ICD and releasing tumor antigens and danger signals. For example, viruses armed with transgenes encoding immune-stimulating factors like GM-CSF, as seen with talimogene laherparepvec, can create a potent *in situ* vaccination effect, recruiting and activating dendritic cells and T cells within the tumor (Zhang Z. et al., 2026). In addition, stimulator of interferon gene agonists directly activate the cGAS-STING pathway in antigen-presenting and tumor cells. This triggers robust type I interferon responses and promotes the cross-priming of tumor-specific CD8^+^ T cells. Advanced nano-delivery systems designed to target these agonists have shown a remarkable ability to inflame cold tumors in preclinical models, with several candidates now advancing into clinical trials (Sagliocchi et al., 2026).

Epigenetic therapies such as DNMTis and HDAC inhibitors, can induce a state of “viral mimicry” by reactivating endogenous retroviral elements. This leads to the sensing of double-stranded RNA and subsequent production of type I interferon, thereby enhancing tumor immunogenicity and promoting T cell infiltration (Ozkerim et al., 2025). Moreover, radiotherapy, particularly hypofractionated or stereotactic body radiotherapy (SBRT), is a potent inducer of ICD and functions as an *in situ* vaccine. When combined with immune checkpoint blockade, it can help can overcome local immunosuppression and potentially generate a systemic anti-tumor immune response (Khan et al., 2025).

Cytokine-based therapies, including IL-15 superagonists and engineered cytokines with extended half-lives, are designed to promote the activation and proliferation of T cell and NK cells. These agents are being evaluated in combination with ICIs to convert immunologically cold tumors (Guo et al., 2025). Bispecific T cell engagers represent another strategy by physically redirecting polyclonal T cells to tumor cells, independent of TCR specificity. While systemic administration can be associated with toxicity, innovative approaches such as intratumoral delivery or the use of conditionally active engagers activated specifically by tumor microenvironment proteases are being developed to drive localized T-cell infiltration and activation (Lipreri et al., 2025). The optimal approach likely involves rational combinations that simultaneously address multiple barriers to T cell infiltration, activation, and persistence. For example, combining an oncolytic virus to induce inflammation with a STING agonist to amplify dendritic cell activation and an ICI to relieve T cell suppression represents a multi-pronged strategy to reprogram cold tumors (Sun G. et al., 2025).

### 5.3 Targeting metabolic and epigenetic pathways

Directly targeting the metabolic and epigenetic wiring of the TME aims to reverse the immunosuppressive landscape and impair tumor-stroma symbiosis.

#### 5.3.1 Metabolic interventions

The goal is to relieve metabolic suppression of immunity and starve tumor-supportive pathways. Strategies include: inhibiting IDO1 to prevent tryptophan depletion and kynurenine production; blocking ARG1 in MDSCs to restore arginine levels for T cells; and targeting lactate dehydrogenase A (LDHA) to reduce lactate-driven acidosis and signaling (Cai et al., 2025; Zhang S. et al., 2026; Xiang et al., 2025). Furthermore, inhibiting FAO, using drugs like etomoxir, can specifically impair the function of intra-tumoral Tregs and disrupt the metabolic activity of meCAFs (Zhu et al., 2026; Bakir et al., 2025).

#### 5.3.2 Epigenetic therapies

Low-dose DNMTis, such as azacitidine and decitabine, along with HDAC inhibitors like vorinostat and entinostat, are being repurposed as TME-modulating agents. At these doses, their primary role is not necessarily direct tumor cells killing, but rather to reverse immune-evasion signatures, induce viral mimicry, and alter the differentiation and function of immunosuppressive cells (Goyal et al., 2023; Wang H. et al., 2025; Xu Y. et al., 2025). Inhibitors targeting other epigenetic regulators, including BET inhibitors that targeting BRD4 and EZH2 inhibitors, are also in clinical trials. These agents aim to disrupt oncogenic transcriptional programs that maintain tumor cell stemness and stromal activation (Feng et al., 2026; Arora et al., 2026).

### 5.4 Stromal-targeted and vascular-targeting therapies

The therapeutic strategy in this area has evolved from non-selective depletion, which can paradoxically worsen disease, toward precise modulation or normalization of the TME components.

#### 5.4.1 CAF reprogramming

Instead of attempting globally elimination of CAFs, the current focus is on targeting specific pathogenic subpopulations or their pro-tumorigenic functions. Strategies include inhibiting the metabolic enzyme CPT1A in meCAFs to disrupt lactate production, deploying agents that target FAP, and blocking downstream signaling of CAF-derived factors, for instance using CXCR4 antagonists to neutralize CXCL12 signaling.

#### 5.4.2 ECM normalization

Degrading the physical barrier can enhance drug penetration. For example, PEGylated recombinant human hyaluronidase enzymatically degrades hyaluronic acid, a major ECM component, thereby reducing interstitial fluid pressure. Inhibitors of enzymes like lysyl oxidase (LOXL2), which are responsible for collagen cross-linking and ECM stiffening, have shown promise in preclinical models (Shen T. et al., 2025).

#### 5.4.3 Vascular normalization

As detailed previously, the judicious use of anti-angiogenic agents, including bevacizumab and sunitinib, at specific lower doses can prune immature vessels and fortify the remaining vasculature. This creates a transient normalization window characterized by improved perfusion and reduced hypoxia, which represents the optimal timing for co-administering chemotherapy or immunotherapy to enhance their efficacy (Rovers et al., 2025).

### 5.5 Microbiome modulation

The gut-tumor axis presents a novel, systemic lever to pull in modulating the TME. Therapeutic approaches are rapidly advancing from correlation to intervention.

#### 5.5.1 Fecal microbiota transplantation (FMT)

The most direct approach, transferring the complete microbial community from an ICI responder to a non-responder, has demonstrated proof-of-concept in re-sensitizing tumors (Elkrief and Routy, 2021; Derosa et al., 2021). In landmark clinical studies, patients with refractory melanoma who received FMT from responding patients showed objective responses to anti-PD-1 therapy that had previously failed, accompanied by favorable changes in immune cell infiltration and gene expression profiles in the TME (Davar et al., 2021).

The mechanisms underlying FMT-mediated TME reprogramming are multifaceted. Beneficial bacterial taxa, such as *Akkermansia muciniphila*, *Faecalibacterium prausnitzii*, *Bifidobacterium* species, produce metabolites including short-chain fatty acids (SCFAs) and inosine. These metabolites enhance dendritic cell maturation, promote Th1 cell differentiation, and support CD8^+^ T cell effector function (El et al., 2024).

Important considerations for clinical application encompass rigorous donor screening to prevent pathogens transmission, standardization the preparation protocol regarding formulation and storage, and optimization of the administration route, with oral capsules generally preferred for convenience and safety. Ongoing clinical trials are actively evaluating FMT in combination with ICIs across multiple cancer types (Barragan-Carrillo et al., 2025).

#### 5.5.2 Defined bacterial consortia

To improve safety, reproducibility, and regulatory compliance, defined bacterial consortia are being developed. There are standardized oral capsules containing curated mixes of beneficial bacterial strains that have been correlated with ICI response. SER-401, a consortium of Ruminococcaceae species developed by Seres Therapeutics, has been evaluated in combination with nivolumab for melanoma (Glitza et al., 2024). Other consortia designed to modulate the gut-tumor axis are in preclinical and early clinical development.

#### 5.5.3 Prebiotics and probiotics

Supplementation strategies also include the use of prebiotics, which are specific dietary fibers that nourish beneficial bacteria, which are live bacterial supplements (Simpson et al., 2023). There represents a more accessible, though potentially less potent, intervention strategy compared to FMT or defined consortia.

### 5.6 Radiation therapy as a TME modulator

Radiotherapy is being re-envisioned not just as a local cytoreductive modality, but as a powerful *in situ* immune modulator. Hypofractionated or stereotactic body radiotherapy (SBRT), which delivers high, ablative doses in few fractions, is particularly effective at inducing ICD. This releases tumor antigens and danger signals, including ATP, calreticulin, and HMGB1, leading to dendritic cell activation and priming of tumor-specific T cells, thereby effectively creating an *in situ* vaccine (Penninckx et al., 2023). When combined with immune checkpoint blockade, radiotherapy can help overcome local immunosuppression and potentially elicit a systemic anti-tumor response, known as the abscopal effect, making it a potent partner in TME-reprogramming combination regimens (Yijun et al., 2025) Figure 3.

**FIGURE 3:**
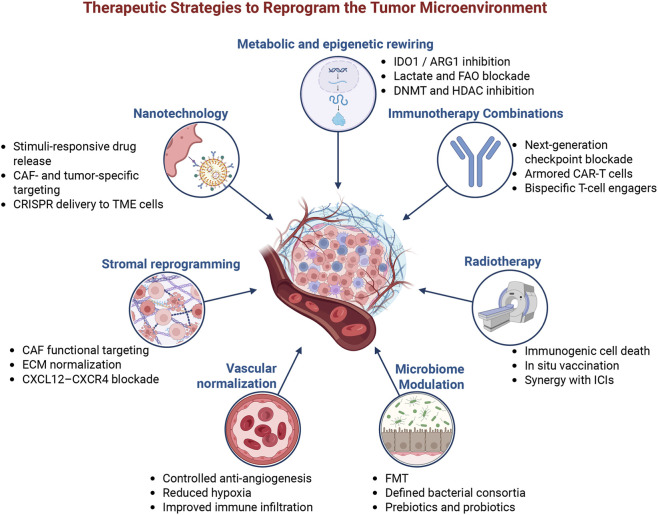
Emerging therapeutic strategies aimed at reprogramming the tumor microenvironment to restore anti-tumor immunity and enhance treatment efficacy. *Multiple intervention axes converge on the TME, targeting its metabolic, epigenetic, immune, stromal, vascular, and systemic regulatory components. Nanotechnology-based platforms enable stimuli-responsive and cell-specific delivery of therapeutics, including gene-editing tools, to overcome physical and biochemical barriers. Metabolic and epigenetic rewiring strategies disrupt tumor–stroma symbiosis and reverse immune suppression through inhibition of immunoregulatory metabolic pathways and chromatin modifiers. Rational immunotherapy combinations, including next-generation checkpoint inhibitors, engineered adoptive cell therapies, and bispecific T-cell engagers, aim to overcome redundant immune-evasion mechanisms. Stromal reprogramming and extracellular matrix (ECM) normalization reduce physical exclusion of immune cells and therapeutics, while vascular normalization alleviates hypoxia and improves immune infiltration. Microbiome modulation represents a systemic approach to tune immune tone and therapy responsiveness. Radiotherapy is re-envisioned as an immune-modulating modality that induces immunogenic cell death and synergizes with immunotherapies. Collectively, these strategies highlight a paradigm shift from direct tumor cell targeting toward ecosystem-level reprogramming of the TME.*

## 6 Clinical translation, challenges, and future perspectives

The translation of TME-targeting strategies from compelling preclinical models to durable clinical benefit represents the paramount challenge in modern oncology. While the therapeutic toolkit is expanding rapidly, the adaptive and heterogeneous nature of the TME presents formidable hurdles that must be systematically overcome to realize the promise of ecosystem-level cancer therapy.

### 6.1 Persistent challenges in translation

#### 6.1.1 Intratumoral and intertumoral heterogeneity

The TME is not a monolithic entity. Significant heterogeneity exists between cancer types, between patients with the same cancer type, and even within different regions of a single tumor (Jin et al., 2025; Chen M. et al., 2025). A one-size-fits-all approach to stromal or immune reprogramming is therefore unlikely to succeed. For instance, a CAF-depleting strategy effective in a densely fibrotic pancreatic tumor may be irrelevant or harmful in a less desmoplastic melanoma. This necessitates robust biomarkers to classify TME subtypes and guide patient stratification.

#### 6.1.2 Therapeutic plasticity and adaptive resistance

The TME is a dynamic, adaptive system with built-in redundancies. Targeting one immunosuppressive pathway often leads to the compensatory upregulation of another; a phenomenon known as adaptive resistance. For example, depletion of M2-like TAMs using CSF-1R inhibitors has, in some trials, led to a compensatory increase in Treg infiltration or upregulation of alternative checkpoint ligands, blunting therapeutic efficacy (Zhou et al., 2022; Lv et al., 2024). This underscores the need for rational, multi-target combinations that preempt escape routes, but designing such regimens is complex.

#### 6.1.3 Biomarker development and patient selection

A major bottleneck is the lack of validated predictive biomarkers to identify which patients will benefit from a specific TME-modulating therapy. Current biomarkers like PD-L1 immunohistochemistry are insufficient. The future lies in integrated signatures: spatial biomarkers from multiplex immunohistochemistry or digital pathology that map immune-stromal relationships; circulating biomarkers like exosomal cargo or T cell receptor repertoires; microbiome profiles from stool samples; and epigenetic signatures from liquid biopsies (Ozkerim et al., 2025; Khan et al., 2025; Lin et al., 2026). Developing and standardizing these assays for clinical use is a critical unmet need.

#### 6.1.4 Delivery and specificity

Achieving sufficient drug concentration within the TME while sparing healthy tissues remains a profound challenge, especially for stromal targets. Many targets on CAFs or TAMs, for example, FAP and CD40, are also expressed, albeit at lower levels, on their healthy counterparts involved in wound healing and homeostasis. Off-target effects can lead to significant toxicity. Nanotechnology and conditionally active biologics, such as probodies and bispecifics activated by TME proteases, offer promising solutions to this delivery dilemma (Deng et al., 2025; Xiong et al., 2026).

#### 6.1.5 Complexity of combination trials

The logical progression towards multi-agent regimens targeting different TME components creates daunting practical challenges. Determining the optimal dosing, sequencing, and schedule for combinations, for instance a vascular normalizer plus an epigenetic drug plus an ICI, requires sophisticated trial design. Furthermore, overlapping and novel toxicities must be carefully managed, and the commercial and regulatory pathways for such complex combinations are not yet fully defined.

#### 6.1.6 Incorporating dynamic TME monitoring into clinical trial designs

The dynamic and adaptive nature of the TME necessitates a shift from static biomarker assessment to longitudinal monitoring within clinical trials. Several innovative trial designs are emerging to address this need.

Adaptive platform trials, such as the I-SPY 2 and FOCUS4 studies, incorporate serial tissue and liquid biopsies to track TME evolution in real time. In the I-SPY 2 trial for high-risk breast cancer, dynamic changes in the density of tumor-infiltrating lymphocytes (TILs), and in PD-L1 expression during neoadjuvant therapy were used to guide subsequent treatment assignments (Valenza et al., 2023). Patients showing increased TILs levels, indicating a more inflamed TME, were randomized to immunotherapy-containing regimens, while those with stable or decreased TILs received alternative combinations.

Monitoring of circulating tumor DNA (ctDNA) and immune cell enables the non-invasive tracking of TME evolution. In a recent phase II trial of pembrolizumab in advanced melanoma, early ctDNA clearance, observed within 3 weeks, predicted treatment response. Conversely, rising ctDNA levels preceded radiographic progression by a median of 8 weeks, allowing for an early switch to salvage therapy. Additionally, monitoring dynamic changes in peripheral immune cell subsets, for example, Ki67+ PD-1+ CD8^+^ T cells, provided a pharmacodynamic readout of systemic immune activation (Amir et al., 2024).

Advanced imaging biomarkers allow for the non-invasive, serial assessment of TME states. These include hypoxia PET using tracers such as ^18^F-FMISO or ^64^Cu-ATSM, and immunoPET targeting molecules like PD-L1, CD8, or granzyme B. In a proof-of-concept study, patients with non-small cell lung cancer receiving chemoradiation plus durvalumab underwent ^89^Zr-durvalumab PET before and during treatment. Increased tumor uptake the tracer correlated with clinical response, while heterogeneous or absent uptake identified patients likely to experience treatment failure (Pouw et al., 2024).

Serial biopsy protocols are being incorporated into window-of-opportunity trials, where patients receive a short course of an experimental agent before definitive surgery. Paired pre- and post-treatment biopsies enable direct assessment of TME modulation, including changes in CAF activation, TAM polarization, and T cell infiltration. For instance, a trial of the CSF-1R inhibitor pexidartinib in breast cancer used serial biopsies to demonstrate successful TAM reprogramming, finding which subsequently informed rational combination trial design (Imani et al., 2025).

Collectively, these approaches enable response-adaptive randomization, where patients are assigned to different treatment arms based on their evolving TME profile, and early escape strategies for patients showing unfavorable TME evolution. Acknowledging this potential, regulatory agencies including the FDA and the EMA have issued guidance encouraging the incorporation of dynamic monitoring in early-phase trials of TME-targeting agents. This is intended to accelerate the identification of active therapeutic regimens and predictive biomarkers (Baldi et al., 2025).

### 6.2 Future perspectives and converging directions

The path forward lies in leveraging technological convergence to address these challenges head-on, moving towards increasingly personalized and dynamic treatment paradigms.

#### 6.2.1 Personalized, multi-modal therapy guided by AI

The future of TME-targeting is inherently personalized. AI will be central, integrating a patient’s unique multi-omics data—encompassing genomics, transcriptomics, and proteomics derived from tumor biopsies—with high-resolution imaging, and microbiome profile to create a digital twin of their TME. AI models will then simulate therapeutic responses to various combination regimens, identifying the bespoke multi-modal strategy most likely to disrupt that specific ecosystem’s vulnerabilities (Khan et al., 2025; Ruan et al., 2025).

#### 6.2.2 Dynamic and adaptive treatment monitoring

Treatment will shift from static protocols to adaptive cycles informed by real-time monitoring. Liquid biopsies tracking circulating tumor DNA (ctDNA) and immune cell dynamics, combined with advanced functional imaging such as hypoxia PET and immunoPET, will allow clinicians to monitor TME evolution in response to therapy (Khan et al., 2025). This enables rapid adaptation, switching or adding therapies if early signs of resistance or phenotypic adaptation emerge.

#### 6.2.3 Next-generation engineering with logic-gated control

Therapeutic agents themselves are evolving towards greater sophistication. The next-generation includes logic-gated CAR-T cells, engineered to activate only upon encountering dual tumor antigens for improved specificity and armored to resist immunosuppression. Oncolytic viruses are being designed with more sophisticated transgene payloads and tighter replication control mechanisms. Concurrently, drug delivery systems are advancing from single-stimuli responsiveness to multi-input logic gates, which release their payloads only when a precise combination of TME conditions, for instance low pH coupled with high matrix metalloproteinase activity and hypoxia, is detected (Rauf et al., 2025; Wu et al., 2026; Tang et al., 2026).

#### 6.2.4 Focus on metastasis prevention

As TME-modulating therapies improve control of primary tumors, a critical Frontier will be targeting the pre-metastatic niche. This involves intercepting the systemic communication via exosomes and bone marrow-derived cells that prime distant organs for metastasis. Prophylactic strategies to disrupt this niche formation could fundamentally alter cancer from a metastatic disease to a locally manageable one. The journey to clinically master the TME is a steep but navigable climb. It demands a departure from reductionist approaches and embraces the complexity of cancer as an ecosystem disease. Through the strategic integration of advanced analytics, sophisticated engineering, and adaptive clinical trial designs, the goal of durably reprogramming the tumor microenvironment; and thereby transforming patient outcomes, is within reach (Yang L. et al., 2025; Rosenthal et al., 2025) Figure 4.

**FIGURE 4:**
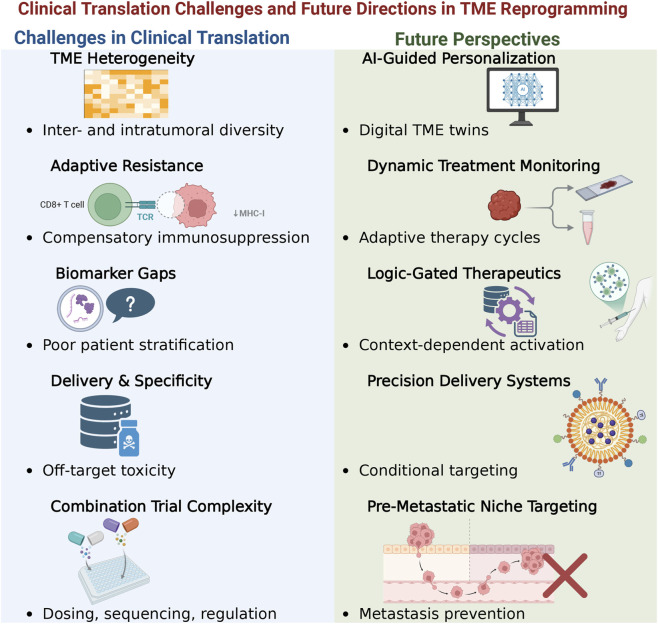
Key challenges and emerging solutions in translating tumor microenvironment.

Major translational barriers include pronounced inter- and intratumoral heterogeneity, adaptive resistance to single-pathway interventions, limited predictive biomarkers, delivery and specificity constraints, and the complexity of multi-agent clinical trial design. Future perspectives highlight converging strategies aimed at overcoming these barriers, including AI-guided personalization of therapy, dynamic treatment monitoring, logic-gated and precision-engineered therapeutics, advanced delivery systems, and targeting of the pre-metastatic niche. Together, these approaches support a shift toward adaptive, ecosystem-level cancer therapies designed to durably reprogram the TME and improve patient outcomes.

## 7 Conclusion

The tumor microenvironment stands as the decisive battlefield in modern oncology. Its complex, adaptive, and co-evolved nature fundamentally underpins the most pressing clinical challenges: metastatic dissemination, therapeutic resistance, and immune evasion. As this review synthesizes, the once-prevailing view of cancer as a disease of autonomous tumor cells has been irrevocably supplanted by an ecological paradigm. This paradigm recognizes that malignant progression is an emergent property of the entire corrupted ecosystem, comprising corrupted stroma, suppressed immunity, and a pathological ECM.

The literature underscores a necessary and profound paradigm shift: truly effective and durable cancer therapy must move beyond a narrow focus on mutational killing of tumor cells to encompass the comprehensive therapeutic reprogramming of the malignant niche. This endeavor is now empowered by an unprecedented convergence of technologies. Nanotechnology provides targeted delivery and smart sensing capabilities; immunotherapy offers tools to reinvigorate and redirect immune effectors; epigenetic drugs can reverse pathological cellular programming; microbiome science reveals a systemic lever for modulation; and advanced analytics, powered by AI and single-cell multi-omics, deliver the necessary resolution to diagnose, model, and predict the behavior of this complex system.

While formidable challenges of intratumoral heterogeneity, therapeutic plasticity, and precise delivery persist, they are not insurmountable. The path forward lies in the strategic, biomarker-driven integration of these multidisciplinary approaches. The future of oncology will be characterized by personalized, multi-modal regimens that simultaneously target the unique vulnerabilities of a patient’s specific TME composition, supported by dynamic monitoring to adapt to ecosystem evolution. By mastering the ecology of the tumor microenvironment, dismantling its support networks, reversing its immunosuppression, and disrupting its adaptive resilience, we hold the definitive key to unlocking durable clinical responses and transforming cancer from a lethal foe into a manageable chronic condition. The mission is clear: to conquer the tumor, we must first conquer its microenvironment.

## Glossary

2D: Two-dimensional
3D: Three-dimensional
α-SMA: Alpha-smooth muscle actin
AhR: Aryl hydrocarbon receptor
AI: Artificial intelligence
APM: Antigen-presenting machinery
apCAF: Antigen-presenting cancer-associated fibroblast
ARG1: Arginase-1
BiTE: Bispecific T cell engager
CAF: Cancer-associated fibroblast
CAR: Chimeric antigen receptor
CARPA: Complement activation-related pseudoallergy
CCL2: Chemokine (C-C motif) ligand 2
CCR2: Chemokine (C-C motif) receptor 2
CD: Cluster of differentiation
circRNA: Circular RNA
CRISPR: Clustered regularly interspaced short palindromic repeats
CSF-1: Colony-stimulating factor 1
CSF-1R: Colony-stimulating factor 1 receptor
ctDNA: Circulating tumor DNA
CTLA-4: Cytotoxic T-lymphocyte-associated protein 4
CXCL12: C-X-C motif chemokine ligand 12
CXCR4: C-X-C chemokine receptor type 4
DNMT: DNA methyltransferase
DNMTi: DNA methyltransferase inhibitor
DTP: Drug-tolerant persister
ECM: Extracellular matrix
EGFR: Epidermal growth factor receptor
EMA: European Medicines Agency
EMT: Epithelial-mesenchymal transition
EndMT: Endothelial-mesenchymal transition
EPR: Enhanced permeation and retention
ERV: Endogenous retrovirus
EV: Extracellular vesicle
EZH2: Enhancer of zeste homolog 2
FAO: Fatty acid oxidation
FAP: Fibroblast activation protein
FDA: Food and Drug Administration
FMT: Fecal microbiota transplantation
GPR81: G-protein coupled receptor 81
HDAC: Histone deacetylase
HER2: Human epidermal growth factor receptor 2
HIF-1α: Hypoxia-inducible factor 1-alpha
HLA: Human leukocyte antigen
ICI: Immune checkpoint inhibitor
ICD: Immunogenic cell death
IDO1: Indoleamine 2,3-dioxygenase 1
IFN: Interferon
iCAF: Inflammatory cancer-associated fibroblast
IL: Interleukin
iNOS: Inducible nitric oxide synthase
LAG-3: Lymphocyte-activation gene 3
LDHA: Lactate dehydrogenase A
LIF: Leukemia inhibitory factor
LNP: Lipid nanoparticle
lncRNA: Long non-coding RNA
LOX: Lysyl oxidase
LOXL2: Lysyl oxidase-like 2
MCT: Monocarboxylate transporter
MDSCs: Myeloid-derived suppressor cells
meCAF: Metabolic cancer-associated fibroblast
MERFISH: Multiplexed error-robust fluorescence *in situ* hybridization
MHC: Major histocompatibility complex
miRNA: MicroRNA
MMP: Matrix metalloproteinase
MRI: Magnetic resonance imaging
MSC: Mesenchymal stem cell
myCAF: Myofibroblastic cancer-associated fibroblast
NK: Natural killer
OV: Oncolytic virus
OXPHOS: Oxidative phosphorylation
PD-1: Programmed cell death protein 1
PDGF: Platelet-derived growth factor
PDGFRβ: Platelet-derived growth factor receptor beta
PD-L1: Programmed death-ligand 1
PDO: Patient-derived organoid
PET: Positron emission tomography
PLGA: Poly(lactic-co-glycolic acid)
ROS: Reactive oxygen species
SBRT: Stereotactic body radiotherapy
SCFA: Short-chain fatty acid
scRNA-seq: Single-cell RNA sequencing
STING: Stimulator of interferon genes
TAA: Tumor-associated antigen
TAM: Tumor-associated macrophage
T cell: T lymphocyte
TCR: T-cell receptor
TEC: Tumor-associated endothelial cell
TGF-β: Transforming growth factor beta
Th1: T helper type 1
TIGIT: T cell immunoreceptor with Ig and ITIM domains
TIL: Tumor-infiltrating lymphocyte
TIME: Tumor immune microenvironment
TLR: Toll-like receptor
TME: Tumor microenvironment
Treg: Regulatory T cell
T-VEC: Talimogene laherparepvec
VEGF: Vascular endothelial growth factor
VISTA: V-domain Ig suppressor of T cell activation
